# Concentration Sensing by the Moving Nucleus in Cell Fate Determination: A Computational Analysis

**DOI:** 10.1371/journal.pone.0149213

**Published:** 2016-02-12

**Authors:** Varun Aggarwal, Richard B. Dickinson, Tanmay P. Lele

**Affiliations:** Department of Chemical Engineering, University of Florida, Gainesville, Florida, United States of America; Baylor College of Medicine, UNITED STATES

## Abstract

During development of the vertebrate neuroepithelium, the nucleus in neural progenitor cells (NPCs) moves from the apex toward the base and returns to the apex (called interkinetic nuclear migration) at which point the cell divides. The fate of the resulting daughter cells is thought to depend on the sampling by the moving nucleus of a spatial concentration profile of the cytoplasmic Notch intracellular domain (NICD). However, the nucleus executes complex stochastic motions including random waiting and back and forth motions, which can expose the nucleus to randomly varying levels of cytoplasmic NICD. How nuclear position can determine daughter cell fate despite the stochastic nature of nuclear migration is not clear. Here we derived a mathematical model for reaction, diffusion, and nuclear accumulation of NICD in NPCs during interkinetic nuclear migration (INM). Using experimentally measured trajectory-dependent probabilities of nuclear turning, nuclear waiting times and average nuclear speeds in NPCs in the developing zebrafish retina, we performed stochastic simulations to compute the nuclear trajectory-dependent probabilities of NPC differentiation. Comparison with experimentally measured nuclear NICD concentrations and trajectory-dependent probabilities of differentiation allowed estimation of the NICD cytoplasmic gradient. Spatially polarized production of NICD, rapid NICD cytoplasmic consumption and the time-averaging effect of nuclear import/export kinetics are sufficient to explain the experimentally observed differentiation probabilities. Our computational studies lend quantitative support to the feasibility of the nuclear concentration-sensing mechanism for NPC fate determination in zebrafish retina.

## Introduction

Mitosis in the proliferating vertebrate neuroepithelium occurs at the apical end of the neuroepithelium [1–8]. The nucleus in the progenitor cell moves from the apex to the base in a persistent fashion [9–11] interspersed with stochastic back and forth movements and nuclear waiting [12, 13]. The nucleus can turn and move persistently back toward the apex at any position [12]. Upon reaching the apex, the cell divides to form two daughter cells. Baye et al [14] observed that in the developing retina of a zebrafish the closer the nucleus gets to the base during its migration, the higher is the probability of the cell dividing in a neurogenic mode to produce one or two daughter neurons, as opposed to a symmetric proliferative mode where division produces two proliferative progenitors.

The mechanism by which nuclear position influences cell fate decisions is attributed to an apico-basal gradient in the Notch cytoplasmic signal [15, 16]. The levels of cytoplasmic Notch intracellular domains (NICD), released from proteolytic cleavage at the apical membrane, are deduced to be higher in the cytoplasm at the apex and lower toward the base based on observations of an accumulation of NICD in apical nuclei [16]. As the nuclei move from the apex to the base, they sample the NICD concentration through the nuclear import/export pathway. At the base where cytoplasmic NICD levels are thought to be low, the nucleus loses its NICD due to preferential export. A drop in the NICD levels in the nucleus decreases the concentration of Her4 (and other homologous genes) which results in a down-regulation of genes that inhibit the differentiation pathway [17]. This mechanism is proposed to result in a nuclear trajectory-dependent probability of differentiation [14, 16].

The exposure of the nucleus to NICD depends on its trajectory and on the nature of the cytoplasmic NICD gradient; the latter has not been experimentally measured in neural progenitor cells (hereafter called NPCs). How a trajectory-dependent probability of neurogenic differentiation at the apex exists despite the complex stochastic motions of the nucleus, which would continuously expose it to varying levels of the NICD concentration, is also not clear, and is a challenge to the nuclear concentration sensing mechanism.

In this paper we performed stochastic simulations of a moving nucleus in spatial gradients of cytoplasmic NICD. We used previously measured velocities of nuclear migration, distributions of waiting times before changes in nuclear direction, the trajectory-dependent probabilities of nuclear turning and nuclear positional fluctuations in the developing retina of the zebrafish[14]. The fluctuating time-dependent Notch concentration in the nucleus was calculated. Mean times for which the Notch dropped below a threshold concentration were used to compute trajectory-dependent probabilities of commitment to neuronal lineage. Comparisons with the experimentally measured trajectory-dependent probabilities and with nuclear NICD levels revealed the requirement of a sharp gradient in the cytoplasmic NICD concentration to explain the observed differentiation probabilities. The model also predicts how the probability of the differentiation is sensitive to the nuclear import/export rates, nuclear motion and the nature of the cytoplasmic gradient.

## Results

### Estimation of the NICD cytoplasmic gradient

NICD is present in the nucleus when it is close to the apex, but is essentially absent when the nucleus is farther away (refer Fig 5 panel J in reference [16]). As the nuclear concentration of NICD depends on the cytoplasmic NICD concentration, this observation suggests the existence of a cytoplasmic gradient of NICD decreasing from the apex toward the base. Assuming the gradient is determined by production of NICD at the apex [18] and cytoplasmic diffusion and degradation throughout the cell length, the nature of the cytoplasmic gradient would depend on the Thiele modulus $\phi^{2}=\frac{kL^{2}}{D}$ (see Materials and Methods) which reflects the rate of cytoplasmic degradation, *k* relative to the rate of cytoplasmic diffusion (diffusion coefficient *D* [19]) of NICD over the length of the NPC (*L*). For reaction diffusion problems of this nature, large values of *ϕ* correspond to steep gradients and vice versa.

**Fig 1:**
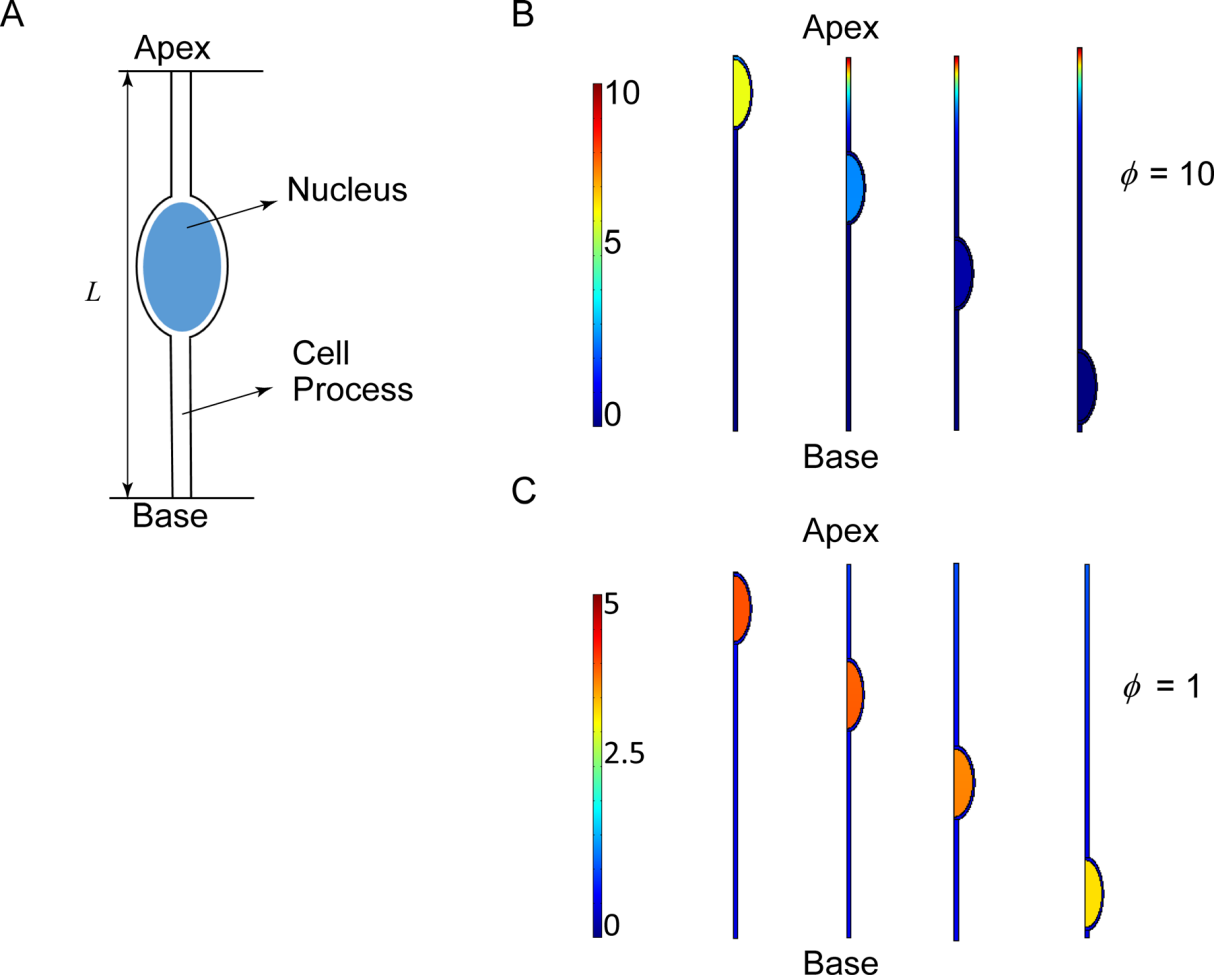
Effect of Cytoplasmic Gradient on Nuclear NICD Levels. A. The geometry of the neural progenitor cell used in the simulations. B. and C. Finite element solutions of the NICD reaction diffusion model for *ϕ* = 10 and *ϕ* = 1 respectively. In B (*ϕ* = 10), the nucleus located at the apex has high levels of NICD, while closer to the base, it has close to zero NICD concentration. The cytoplasmic concentration remains more or less the same. In C, (*ϕ* = 1) the NICD concentration in the nucleus fails to fall to negligible levels, even when the nucleus is very close to the base.

We modeled the reaction-diffusion of NICD and its nuclear import/export in order to compute the NICD cytoplasmic gradient in a geometry characteristic of the retinal NPC (Fig 1A; see Materials and Methods for model details). We estimated the dimensions of a cell length and nucleus (assumed to be an ellipsoid) from microscopic images of retinal neuroepithelium in Ref. [16] (Table 1). Motivated by observations by Clark et al that the size of the apical domain primarily determines the extent of Notch signaling, NICD was assumed to be primarily generated at the apical end [18].

**Table 1:** Model Parameters.

Parameter	Symbol	Value	Source
Cell width	*w*	1 μm	Measured from images taken from [16]
Cell length	*L*	50 μm	Measured from images taken from [16]
Nuclear major axis	*l*	10 μm	Measured from images taken from [16]
Nuclear minor axis		5 μm	Measured from images taken from [16]
Distance between the nuclear membrane and the proximal cell membrane		1 μm	Measured from images taken from [16]
Velocity of nucleus	*v*	8 μm/h (going from apex to base),-27 μm/h (going from base to apex)	Taken from [14]
Cytoplasmic NICD diffusion coefficient	*D*	2.9 μm^2^/s	Taken from [19]
NICD cytoplasmic degradation constant	*k*	varied	
NICD nuclear import mass transfer coefficient	*K*_*i*_	12 μm/h	Estimated from [20] (see supporting information)
NICD nuclear export mass transfer coefficient	*K*_*e*_	2 μm/h	Estimated from [21] (see supporting information)
Diffusivity used to model random motion of the nucleus (Wiener process)	*D*_*n*_	0.125 μm^2^/h	Estimated from experimental trajectories [14]

Shown in Fig 1B and 1C are finite element calculations (using COMSOL, see materials and methods) of cytoplasmic concentration profiles and corresponding concentrations of NICD in the moving nucleus for two values of the Thiele modulus, *ϕ* = 10 and *ϕ* = 1. The only other parameters in the calculation are the nuclear import and export rates and speeds of nuclear motion, which were estimated or known from literature [14, 20, 21]; see materials and methods and supporting information. For *ϕ* = 10 corresponding to rapid NICD degradation in the cytoplasm, there is a clear change in nuclear NICD concentration from a high nuclear concentration at the apex to a low nuclear concentration at the base (Fig 1B), consistent with published experimental observation (refer Fig 5 panel J in reference [16]). However, for slower degradation (*ϕ* = 1), NICD is present in the nucleus at high concentrations throughout its trajectory (Fig 1C). These results demonstrate that a steep spatial cytoplasmic gradient created by rapid degradation of NICD is sufficient to explain the observed dependence of nuclear NICD concentration with nuclear position.

### Stochastic sampling of the NICD concentration by the nucleus

**Fig 2:**
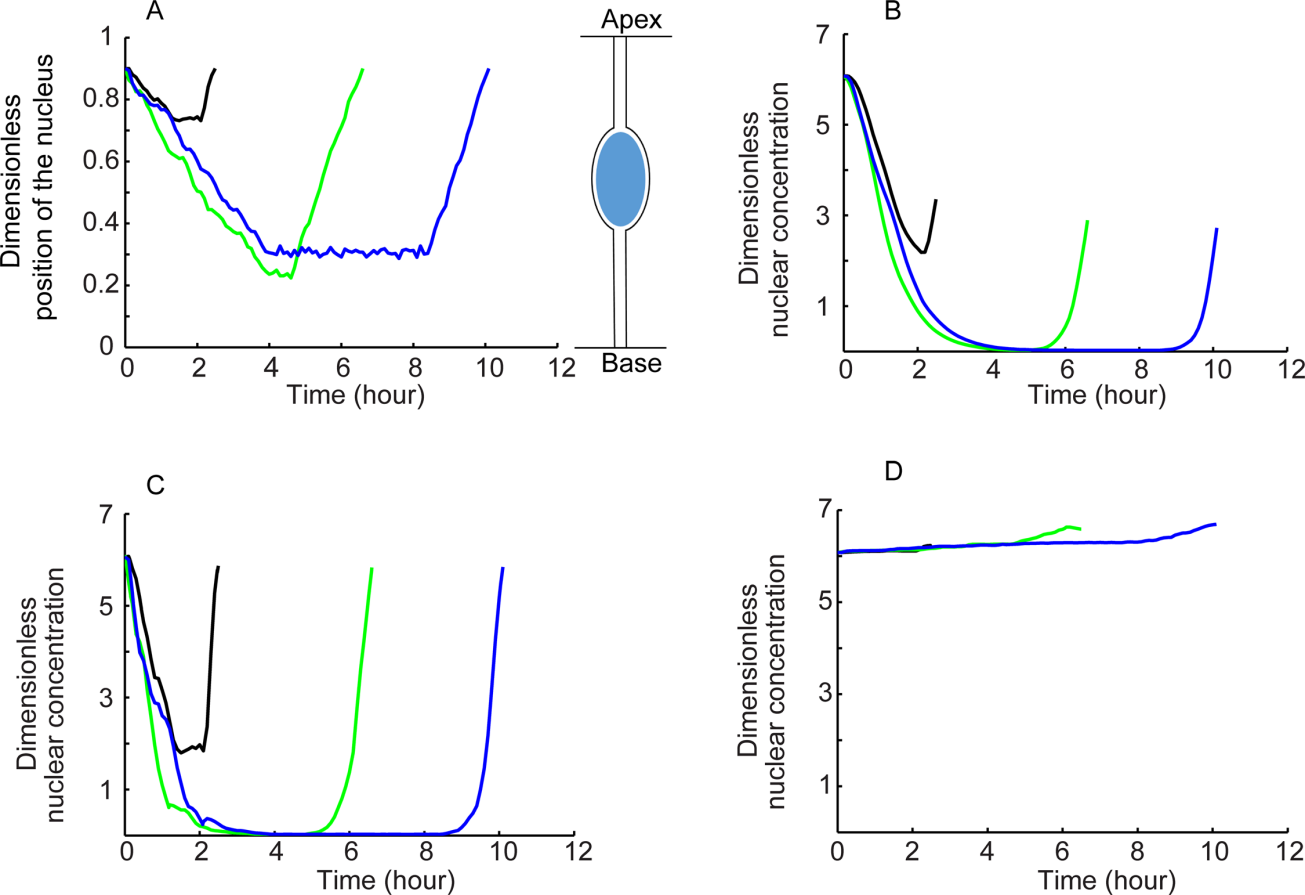
Generation of Nuclear Trajectory and Estimation of Nuclear NICD Concentration. A. Three nuclear trajectories generated by Monte Carlo simulation. B. Nuclear NICD concentration corresponding to trajectories shown in A, showing smooth drop followed by a smooth rise in NICD levels in the nucleus; the dampening is due to import/export kinetics. C. Corresponding nuclear concentrations are plotted for the hypothetical case of instant equilibrium; fluctuations in nuclear trajectory are reflected in the nuclear concentration. D. Nuclear concentration for the hypothetical case of a very low import/export rate; nuclear concentration does not fall and rise in the migrating nucleus.

The translating and fluctuating position of the nucleus was simulated by a stochastic differential equation that accounts for a constant drift velocity as well as position fluctuations of the nucleus. The experimental trajectories of the nucleus exhibit three distinct episodes: a persistent motion away from the apex, a pause, and persistent return to the apex. The experimentally observed distributions[14] of trajectory-dependent probability of turning, distributions of pause times, the drift velocities and positional fluctuations of the nucleus (the latter was used to estimate a diffusion coefficient, Table 1) were used to simulate the trajectory of the nucleus (three example trajectories are shown in Fig 2A). The reaction-diffusion model was solved for *ϕ* = 10 and literature values of import and export rates, as this reproduced the spatial dependence of nuclear NICD levels (discussed above, Fig 1; also refer Fig 5 Panel J in reference [16]).

Shown in Fig 2B are time dependent nuclear NICD concentration profiles corresponding to the nuclear trajectories shown in Fig 2A. The concentration decreases in the nucleus as the nucleus moves from the apex to the base, and later increases when the nucleus turns back toward the apex. As seen in Fig 2B, the fluctuations in nuclear concentrations are damped relative to the fluctuations in the nuclear position owing to the relatively slow nuclear import/export time scales. In contrast, for the hypothetical case of instant transport of NICD between the nucleus and the cytoplasm (Fig 2C), fluctuations in nuclear NICD concentration closely mirror the fluctuations in nuclear position in the NICD gradient. Expectedly, nuclear concentrations remain more or less constant during nuclear motion for (hypothetical) low import/export rates (Fig 2D).

### Position dependence of cell fate decisions

Drop in NICD levels in the nucleus causes a drop in the expression of Her4 and related genes that inhibit the differentiation pathway, resulting in an increased probability of differentiation [17]. As the NICD levels change dynamically during the motion of the nucleus from the apex to the base and back, we hypothesized that the trajectory-dependent probability of differentiation is determined by the time for which NICD is below a threshold level (assumed to be a fraction of the maximum cytoplasmic concentration). We computed this time as a function of the turning position and calculated the mean time among an ensemble of realizations of stochastic nuclear trajectories (hereafter called “mean depletion time”). We calculated the average of the mean depletion times over binned intervals of turning positions (the binned intervals were the same as reported in the experiments[14]), and normalized them to the largest time to compute the probability of differentiation as a function of turning positions.

**Fig 3:**
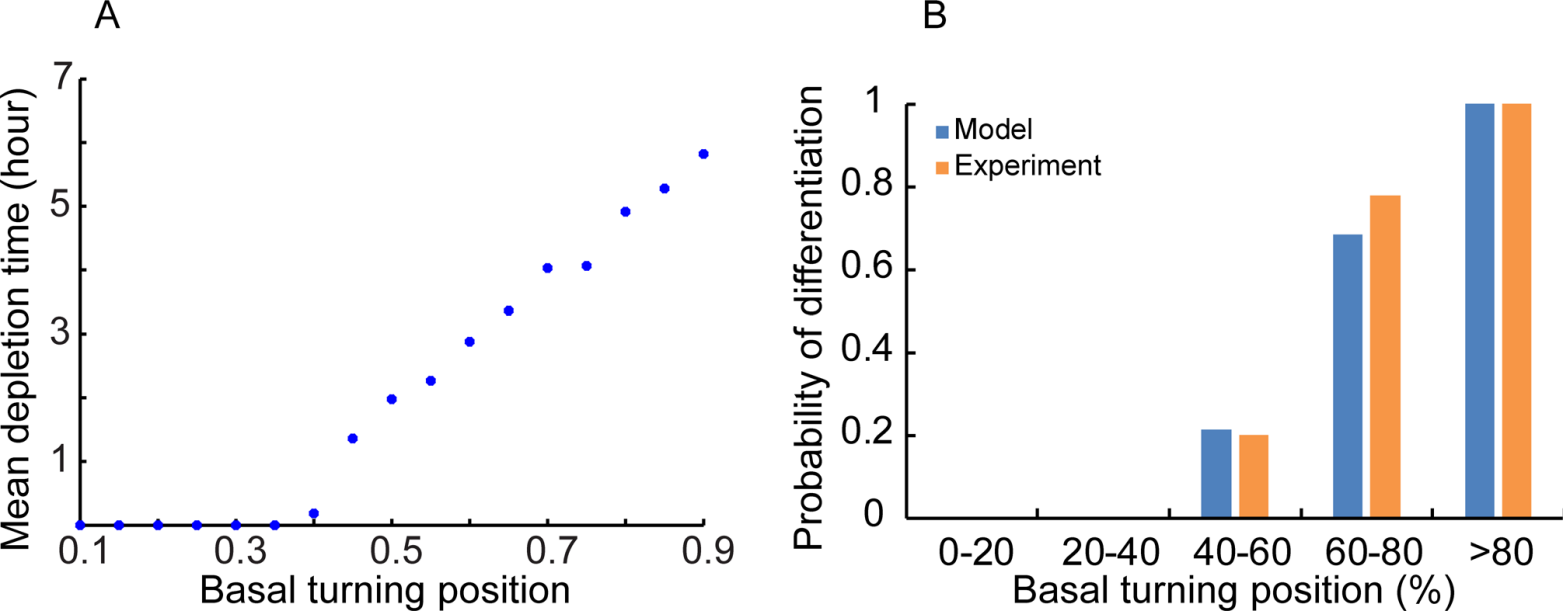
Calculation of Mean Depletion Time and Probabilities. A. Mean depletion time plotted with basal turning positions. B. The bar plot shows the probability of differentiation with basal turning position and comparison with experimental data (experimental data taken from [14]).

There are two unknown parameters in the simulation-*ϕ* and the NICD threshold value. Out of these, *ϕ* must be substantially greater than 1 to allow spatially varying levels of NICD in the nucleus (Fig 1). We explored the sensitivity of the computed spatially dependent probabilities to both *ϕ* and the NICD threshold value (S1 Fig). We found that the computed probabilities increase monotonically with the basal turning position as found in the experiments[14] for a range of parameter values (S1 Fig). Also, there is a reasonable match with experimental values for some parameter sets (Fig 3 shows results for *ϕ* = 10 and NICD threshold = 0.6).

For a given NICD threshold value, the trajectory-dependent probability of differentiation depends on the Thiele modulus. For small values of *ϕ*, there is no differentiation at all (as NICD never falls below the threshold during nuclear motion) while for very large values of *ϕ* the model predicts unrealistically early differentiation (S2 Fig) owing to depletion of nuclear NICD near the ape*x*.

## Discussion

The motion of the nucleus from the apex to the base and back is a remarkable mechanical feat that NPCs accomplish between successive cell divisions in the neuroepithelium. This interkinetic nuclear migration occurs in a diverse range of tissues and across species [22–24], although its function has remained obscure in different cell types. It is known that defects in nuclear motion in NPCs cause excessive early differentiation after mitosis, leading to an abnormal population of differentiated cells and a dysfunctional abnormal retina in zebrafish [16, 25]. In NPCs, nuclear motion has been suggested to control cell fate by nuclear sampling of an apico-basal NICD gradient. In this paper, we propose a computational reaction-diffusion model to explain how nuclear sensing of the NICD concentration by import/export can result in a trajectory-dependent probability of differentiation.

Implicit in our analysis is the assumption that the probability of differentiation for the NPC is proportional to the time for which the nucleus is deprived of NICD, that is, the time for which the NICD level falls below a chosen threshold value. The calculation appears to work well for predicting experimentally observed probabilities and is robust to the choice of the unknown threshold parameter which increases our confidence in the predictions. The longer the time for which NICD levels are low, the lower the Her4 repressor concentrations should be and this will result in a higher probability of differentiation (reviewed in [17]). New experimental information on the kinetic dependence of Her4 up-regulation on NICD levels in NPCs will render the ‘threshold parameter’ in these simulations unnecessary and improve the predictive power of these simulations.

The other unknown parameter in the simulations is the value of the Thiele modulus. Measurements of spatial gradients of the low levels of cytoplasmic NICD are unavailable in the literature. Because the import/export kinetics are known, we were able to provide estimates of the spatial NICD gradient in this paper which produce nuclear NICD close to the apex and rapid depletion farther away (Fig 1) as has been observed in the experiments (refer Fig 5 panel J in reference[16]). We predict relatively sharp NICD gradients to exist in the cytoplasm for the nuclear concentration-sensing mechanism to work.

The stochastic simulations demonstrate that despite the experimentally observed stochastic motion of the nucleus and the relatively small length of the cell (cell length ~ 50 microns compared to a nuclear size of ~10 microns), the nuclear concentration sensing mechanism can yield trajectory-dependent probabilities of neuronal differentiation for experimentally derived parameters. The relatively slow nuclear import/export rates dampen the fluctuations in nuclear NICD concentrations and confer robustness to the sensing mechanism.

In summary, we have tested the feasibility of a nuclear concentration sensing mechanism for explaining cell fate decisions in the developing neuroepithelium. Our results suggest that for a broad range of parameters, it is possible to have a position dependent probability. Future studies that measure the kinetic dependence of Her4 on NICD levels, and the cytoplasmic concentration can further bring clarity to this mechanism.

## Materials and Methods

### Calculation of cytoplasmic gradient

We modeled the transport of the NICD in the cytoplasm and its uptake by the nucleus with a simple reaction diffusion model. The cell geometry is shown in Fig 1A. Using the experimentally determined values of *v*, *L* and *D* (here *v* is the velocity of the nucleus, *L* the length of the cell, and *D* is the diffusion coefficient, see Table 1 for the values), we calculated the Peclet number ($Pe=\frac{vL}{D}$), which was of the order of 0.1. Therefore we neglected convective terms, and the resulting reaction diffusion model could be written as,

$$\frac{\partial C_{c}}{\partial t}=D\nabla^{2}C_{c}-kC_{c}$$ (1)

$$\frac{\partial C_{n}}{\partial t}=D\nabla^{2}C_{n}$$ (2)

Eq 1 describes the reaction diffusion model in the cytoplasm, and Eq 2 describes the model for the nucleus. Here *C*_*c*_ is the concentration of NICD in the cytoplasm, *C*_*n*_ is the concentration in the nucleus, and *k* is the rate constant describing the degradation of NICD. We assumed that NICD is produced only at the apical surface at a constant rate, R [18]. The corresponding boundary conditions are as follows (see S3 Fig for model geometry).

$$-D\frac{\partial C_{c}}{\partial z}=R\;\text{at}\;z=0$$ (3)

$$-D\frac{\partial C_{c}}{\partial z}=0\;\text{at}\;z=L$$ (4)

$$-D\underline{\nabla }C_{c}\cdot \underline{n}=0\;\text{over}\;\Lambda$$ (5)

Here $\underline{n}$ is the vector normal to the cell surface and Λ is the cell boundary.

Nuclear uptake of NICD is described by the following set of equations.

$$-D\underline{\nabla }C_{c}\cdot \underline{n'}=-D\underline{\nabla }C_{n}\cdot \underline{n'}=K_{i}C_{c}-K_{e}C_{n}\;\text{over}\;\Omega$$ (6)

Here $\underline{n'}$ is the vector normal to the nuclear surface, *K*_*i*_ is the import mass transfer coefficient, *K*_*e*_ is the export mass transfer coefficient and Ω is the nuclear surface.

### Motion of the nucleus

The motion of the nucleus consists of linear runs interspersed with random fluctuations, and waiting periods at turning positions. The position of the nucleus was simulated as a stochastic process consisting of a constant drift velocity *v* and a weighted Wiener process $\sqrt{2D_{n}}W_{t}$ such that

$$dz_{n}\left(t\right)=vdt+\sqrt{2D_{n}}dW_{t}$$ (7)

Here *z*_*n*_ is location of the centroid of the nucleus, *v* is nuclear velocity (*v* = 8 μm/hr for the apex-base movement and *v* = 27 μm/hr for the base-apex movement [14]) and *D*_*n*_ is the diffusivity of the Wiener process(*W*_*t*_). The full trajectory of the nucleus was generated using a Monte-Carlo simulation as will be described later. The length of the cell (*L*) was chosen as the length scale, $C_{o}=\frac{R}{kL}$ as the concentration scale and $t_{d}=\frac{L^{2}}{D}$ as the time scale for non dimensionalizing the equations. The non dimensionalized equations are

$$\frac{\partial \theta_{c}}{\partial t_{n}}=\nabla^{2}\theta_{c}-\phi^{2}\theta_{c}$$ (8)

$$\frac{\partial \theta_{n}}{\partial t_{n}}=\nabla^{2}\theta_{n}$$ (9)

here, $\theta_{c}=\frac{C_{c}}{C_{o}}$, $\theta_{c}=\frac{C_{n}}{C_{o}}$, $t_{n}=\frac{t}{t_{d}}$, and $\phi^{2}=\frac{kL^{2}}{D}$. Dimensionless boundary conditions are

$$-\frac{\partial \theta_{c}}{\partial \xi }=\phi^{2}\;\text{at}\;\xi =0$$ (10)

$$-\frac{\partial \theta_{c}}{\partial \xi }=0\;\text{at}\;\xi =1$$ (11)

$${\left(-\underline{\nabla }\theta_{c}\cdot \underline{n}\right)}_{\Lambda }=0$$ (12)

(−∇_θc.n_')Ω=(−∇_θn⋅n'_)Ω=αiθc−αeθn (13)

Here $\xi =\frac{z}{L}$, $\alpha_{i}=\frac{K_{i}L}{D}$, $\alpha_{e}=\frac{K_{e}L}{D}$

### Generation of nuclear trajectories

In accordance with the non-dimensionalizing scheme Eq 7 was modified to

$$d\xi_{n}\left(t\right)=v_{n,nd}dt+\sqrt{2D_{n,nd}}dW_{t}$$ (14)

Here *ξ*_*n*_ is the normalized nuclear position, *v*_*n*,*nd*_ is the normalized velocity, and *D*_*n*,*nd*_ is the normalized diffusivity, all normalized by the length scale. Eq 14 with the appropriate choice of velocity for the corresponding phase of nuclear motion, was used to describe the motion of the nucleus as shown later (see S1 Movie for nuclear motion).

### Calculation of mean depletion time

The position where the nucleus pauses and waiting time of the pause are random variables in the simulations and were calculated from the corresponding probability distributions measured in [14]. Eq 14 was used to generate a trajectory until the basal pause position was reached, at which point the velocity was set to zero for the duration of the pause. The nucleus was moved back to the apex using the corresponding velocity. Around 1500 nuclear trajectories were generated. Corresponding time-dependent nuclear concentrations for each trajectory were calculated using COMSOL. The total time for which the concentration fell below a specified threshold was recorded. This time, called the “depletion time”, depended strongly on the pause position. To compare with the experimental data, the depletion time from the simulations was averaged over intervals of the pause positions to yield values for the mean depletion time, 〈*τ*〉_*i*_, for each interval *i*. For an ensemble of trajectories, 〈*τ*〉_*i*_, is an increasing function of the pause positions in interval *i*. According to the experiments [14] the probability of differentiation approaches one for nuclei that translate the full length of the cell, where 〈*τ*〉_*i*_, is at a maximum. Hence, we assigned the probability of differentiation as

$$p_{i}=\frac{{\langle \tau \rangle }_{i}}{\underset{j}{\text{max}\;{\langle \tau \rangle }_{j}}}$$ (15)

### Estimation of model parameters

The dimension of the cell and the nucleus were determined by measurement from the images taken from [16]. Published values of the velocity of the nucleus [14] and diffusion coefficient of NICD [19] were used for simulation. Parameter values are provided in Table 1.

The value of nuclear NICD import mass transfer coefficient (*K*_*i*_) was estimated using the values published in [20], and nuclear NICD export mass transfer coefficient *K*_*e*_ was estimated using the values published in [21] by employing a mass balance argument (see supporting information). Diffusivity of the Wiener process was chosen to ensure qualitative agreement between simulation trajectories and experimental trajectories. The rate of cytoplasmic NICD degradation was varied as a model parameter to qualitatively reproduce the experimental observation of undetectable nuclear NICD at the basal surface (refer Fig 5 panel J in reference [16]).

### Finite element calculations

COMSOL 4.4 was used to generate a time-dependent nuclear concentration profile. We used transport of dilute species (chds) module to solve for the spatially dependent nuclear and cytoplasmic concentration for a particular nuclear position. Nuclear trajectory was generated in MATLAB 2013 and was supplied to the moving mesh (ale) module which was used to move the nucleus while preserving the overall cell shape (two cylindrical cellular processes and an ellipsoidal cell body). Automatic remeshing was used to regenerate the mesh if the quality of the mesh deteriorated beyond acceptable limits.

COMSOL 4.4 with MATLAB (COMSOL MATLAB Live Link) was used to connect MATLAB and COMSOL. Around 1500 trajectories were generated in MATLAB which were supplied to COMSOL for generating time-dependent concentration profiles. Corresponding spatially averaged time-dependent nuclear concentrations were calculated from COMSOL in the manner described above and were then used in conjunction with the nuclear trajectories to generate mean depletion time versus basal turning position curves. These were subsequently used to calculate trajectory dependent probabilities of differentiation.

## Supporting Information

S1 Fig Probability of Differentiation for Different *ϕ* and Threshold Values. Probability of differentiation for different *ϕ* and threshold values calculated from simulation, and compared with experimental probabilities from reference [14]. The dashed line marks the experimental measurement.
(TIF)

S2 Fig Probability Calculation for *ϕ* = 50. A. Mean depletion time plotted with basal turning position. B. Bar plot showing that differentiation occurs early (20–40% basal turning position) contrary to experiments.
(TIF)

S3 Fig Model Geometry. (TIF)

S4 Fig The Simulated Nuclear Trajectory Corresponding to S1 Movie. (TIF)

S1 Movie Motion of the Nucleus. Movie shows the simulated motion of the nucleus towards the base, stochastic basal pause and return. The corresponding trajectory is show in S4 Fig.
(AVI)

S1 Text Initialization of the Model and Determination of Mass Transfer Coefficients. (DOCX)
