# Supplementary figures and images for: Concentration Sensing by the Moving Nucleus in Cell Fate Determination: A Computational Analysis
Source: PLoS One. 2016 Feb 12;11(2):e0149213. doi: 10.1371/journal.pone.0149213 (PMC4752345; doi:10.1371/journal.pone.0149213)

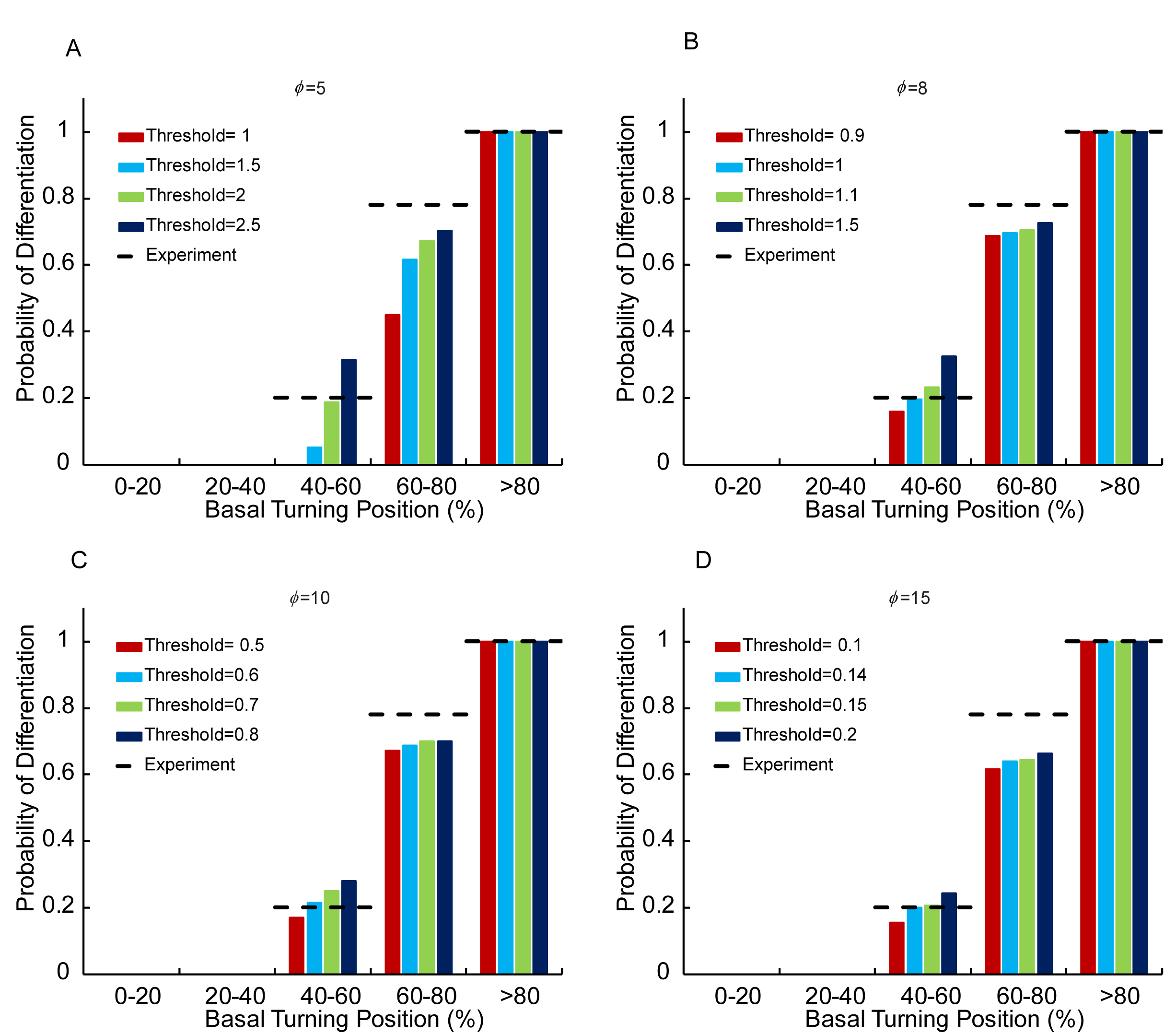

Supplement: S1 Fig — Probability of differentiation for different ϕ and threshold values calculated from simulation, and compared with experimental probabilities from reference [14]. The dashed line marks the experimental measurement. (TIF) [file pone.0149213.s001.tif]

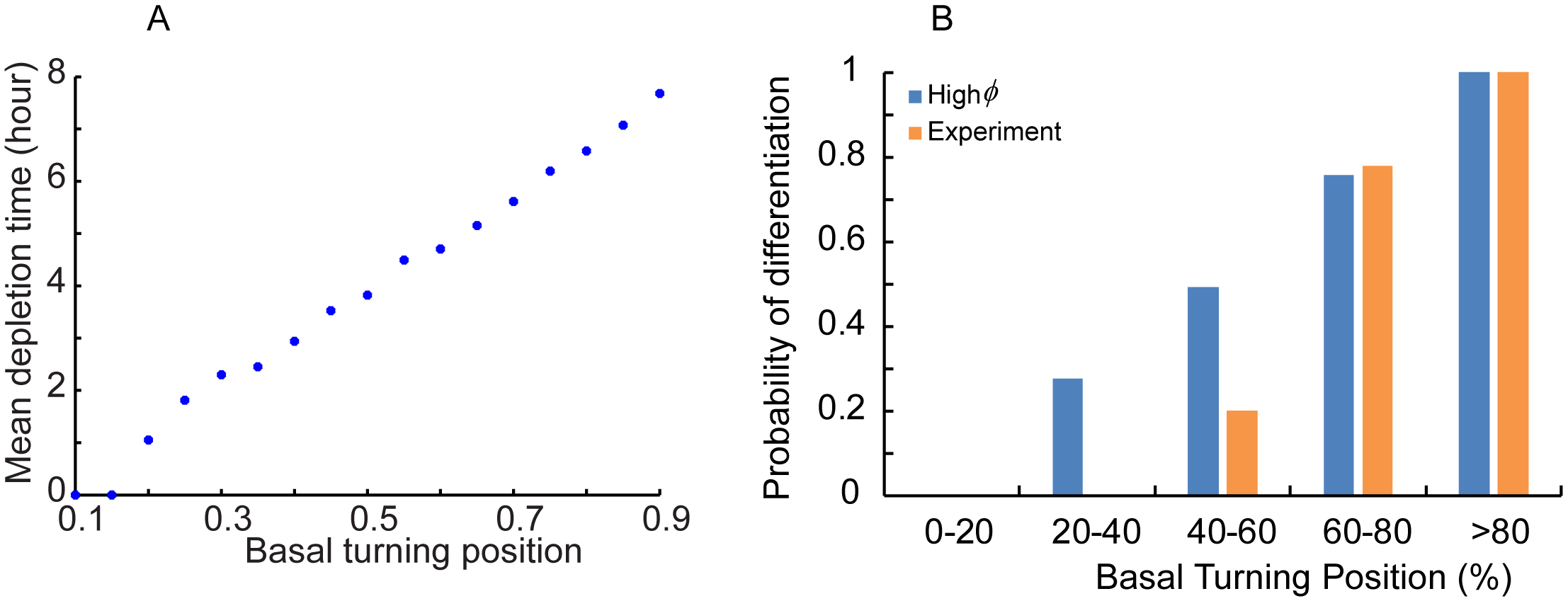

Supplement: S2 Fig — A. Mean depletion time plotted with basal turning position. B. Bar plot showing that differentiation occurs early (20–40% basal turning position) contrary to experiments. (TIF) [file pone.0149213.s002.tif]

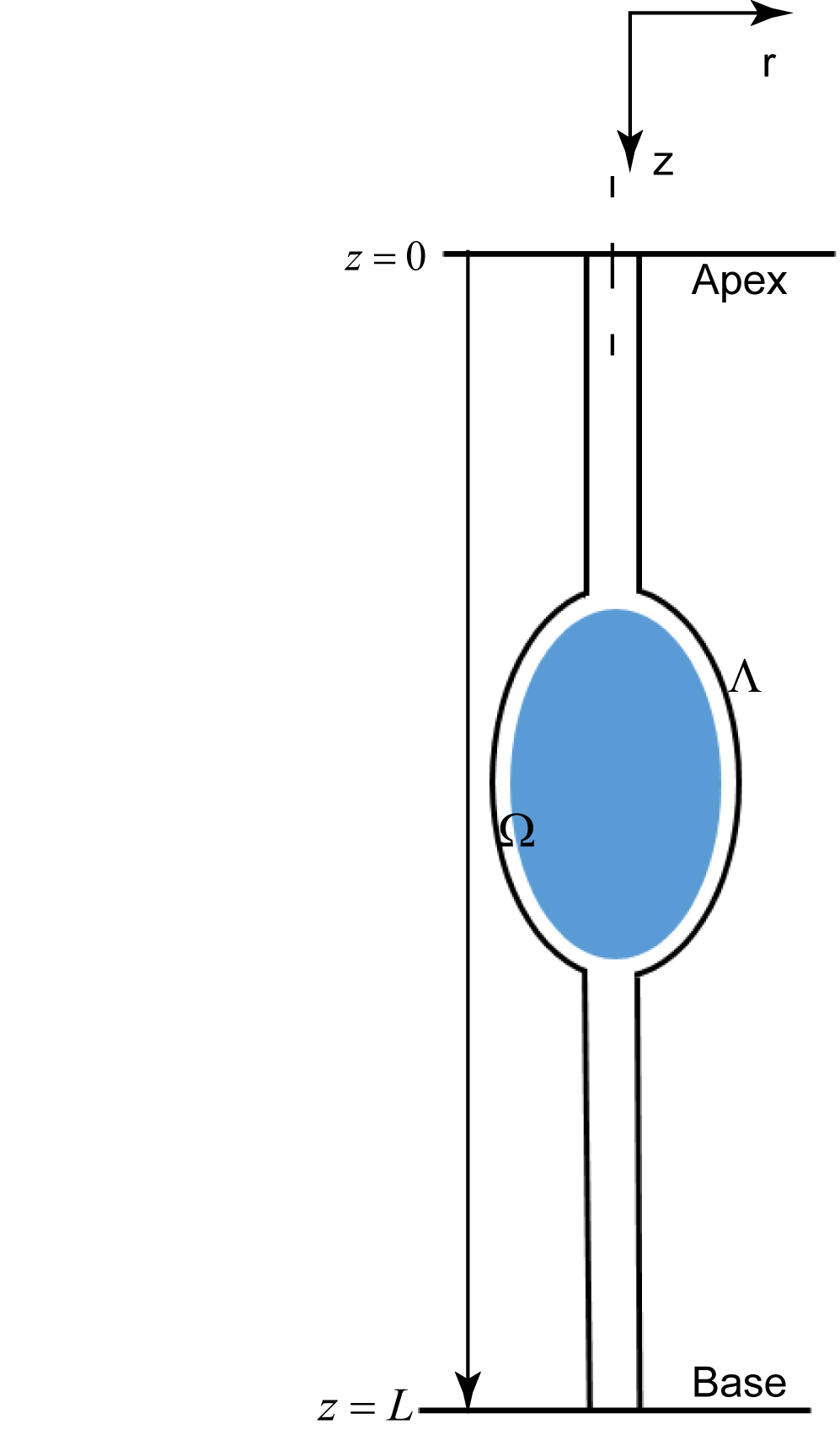

Supplement: S3 Fig — (TIF) [file pone.0149213.s003.tif]

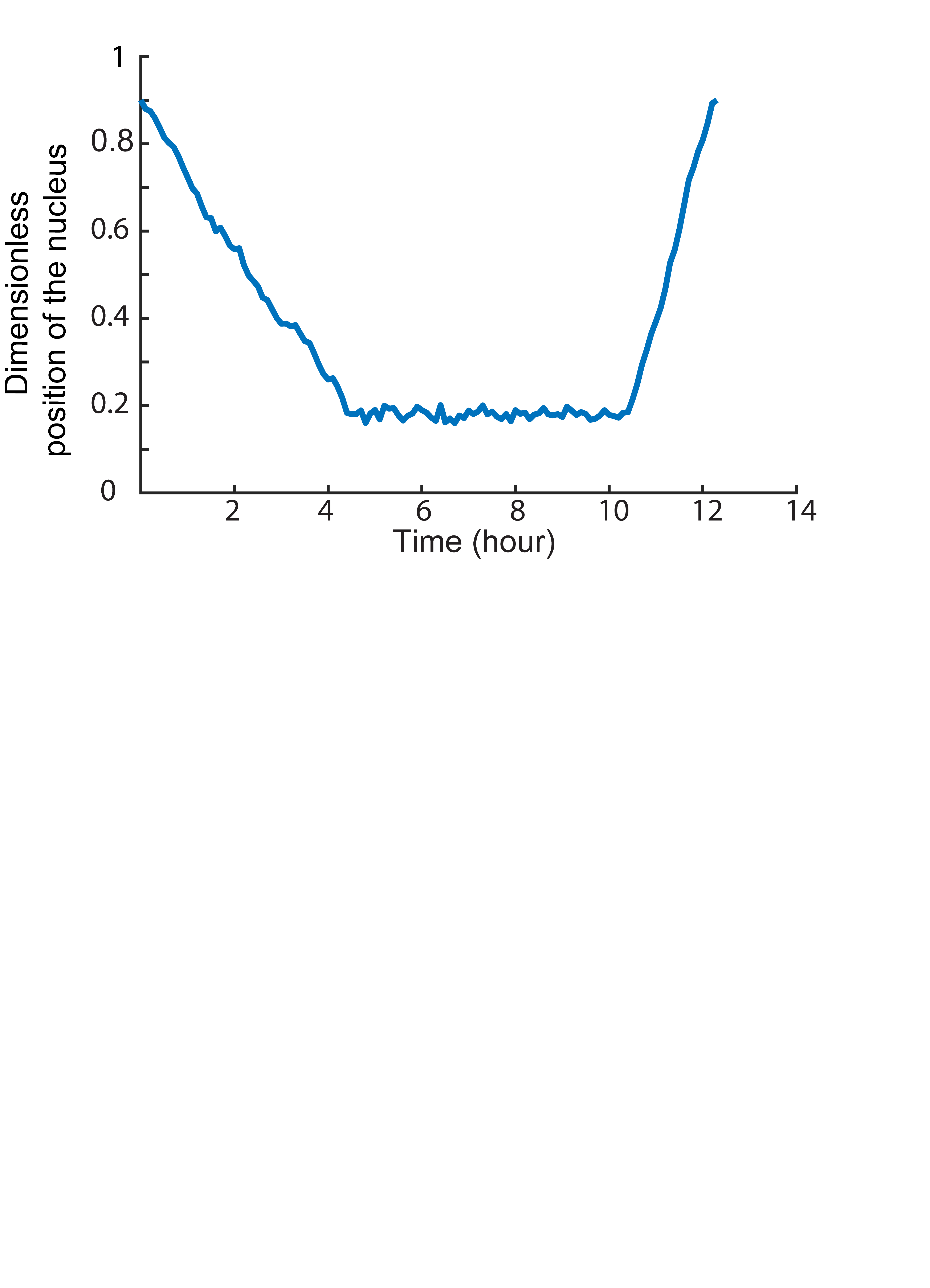

Supplement: S4 Fig — (TIF) [file pone.0149213.s004.tif]
