# Supplementary material for: Concentration Sensing by the Moving Nucleus in Cell Fate Determination: A Computational Analysis
Source: PLoS One. 2016 Feb 12;11(2):e0149213. doi: 10.1371/journal.pone.0149213 (PMC4752345; doi:10.1371/journal.pone.0149213)
Supplement: S1 Text — (DOCX) [file pone.0149213.s006.docx]

**Supporting information**

**Initialization of the Model**

The nucleus was placed at the apex and a steady state nuclear and cytoplasmic concentration was obtained by solving the model in COMSOL. The resulting nuclear and cytoplasmic concentrations were used as the initial nuclear and cytoplasmic concentrations for the calculations with the moving nucleus.

**Determination of Mass Transfer Coefficient from FRAP Data**

The nuclear import/export rate constants reported in literature [1, 2] measured from FRAP experiments were converted to mass transfer coefficients. This was done by a simple mass balance as shown below

| $-D\underline{\nabla}C_{n}\cdot\underline{n'}=K_{i}C_{c}-K_{e}C_{n}$ over $\Omega$ | (S1) |
| --- | --- |

Eq. S1 describes import and export of NICD into the nucleus. Here$D$is the diffusion coefficient of NICD,$C_{n}$ is the concentration of NICD in the nucleus, $\underline{n'}$ is the vector normal to the nuclear surface, $K_{i}$and $K_{e}$are import and export mass transfer coefficients respectively and $\Omega$ is the nuclear surface.

Under the assumption of slow consumption in the nucleus the net rate of change of the concentration in the nucleus is equal to the net influx into the nucleus. Integrating S1 over the nuclear area yields

| $\frac{V_{n}dC_{n}}{dt}=\int_{A_{n}} \left(K_{i}C_{c}-K_{e}C_{n}\right)dA$ | (S2) |
| --- | --- |

Here $A_{n}$is the area of the nucleus and $V_{n}$ is the volume of the nucleus. Under the assumption of uniform nuclear and cytoplasmic concentration, eq. S2 reduces to

| $\frac{dC_{n}}{dt}=\frac{K_{i}A_{n}}{V_{n}}C_{c}-\frac{K_{e}A_{n}}{V_{n}}C_{n}$ | (S3) |
| --- | --- |

FRAP data is typically modeled as

| $\frac{dC_{n}}{dt}=k_{i}C_{c}-k_{e}C_{n}$ | (S4) |
| --- | --- |

$k_{i}$and $k_{e}$are FRAP rate constants.

Comparing S4 and S3 we get:

| $K_{i}=\frac{k_{i}V_{n}}{A_{n}}$ | (S5) |
| --- | --- |
| $K_{e}=\frac{k_{e}V_{n}}{A_{n}}$ | (S6) |

S5 and S6 are the relationship between FRAP rate constants and mass transfer coefficients. $V_{n}$ and $A_{n}$were calculated assuming that the nucleus is an ellipsoid and using the measured value of nucleus major axis and minor axis (measured from images taken from [3]).

**Supporting References**

1. Agrawal S, Archer C, Schaffer DV. Computational models of the Notch network elucidate mechanisms of context-dependent signaling. PLoS Comput Biol. 2009;5(5):e1000390. doi: 10.1371/journal.pcbi.1000390. PubMed PMID: 19468305; PubMed Central PMCID: PMCPMC2680760.

2. Cardarelli F, Tosti L, Serresi M, Beltram F, Bizzarri R. Fluorescent recovery after photobleaching (FRAP) analysis of nuclear export rates identifies intrinsic features of nucleocytoplasmic transport. J Biol Chem. 2012;287(8):5554-61. doi: 10.1074/jbc.M111.304899. PubMed PMID: 22190681; PubMed Central PMCID: PMCPMC3325589.

3. Del Bene F, Wehman AM, Link BA, Baier H. Regulation of neurogenesis by interkinetic nuclear migration through an apical-basal notch gradient. Cell. 2008;134(6):1055-65. doi: 10.1016/j.cell.2008.07.017. PubMed PMID: 18805097; PubMed Central PMCID: PMCPMC2628487.
